# The Distinct Roles of Personal and Perceived School Intelligence Mindsets in Learning Motivation and Achievement

**DOI:** 10.3390/jintelligence14050081

**Published:** 2026-05-09

**Authors:** Kai Zhang, Wu-jing He, Hui-fei Jiang

**Affiliations:** 1 Department of Special Education & Counselling, The Education University of Hong Kong, Hong Kong SAR, China; kaizhan126@gmail.com; 2 School of Education, The University of New South Wales, Sydney, NSW 2502, Australia; huifeijiang@unsw.edu.au

**Keywords:** perceived school intelligence mindset, personal intelligence mindset, learning motivation, academic achievement, mediation, self-determination theory

## Abstract

From the perspective of self-determination theory (SDT), this study investigated (1) how a perceived school intelligence mindset (PSIM) functions as an antecedent of a personal intelligence mindset (PIM) and (2) how these two belief systems differentially predict students’ learning-related outcomes. A total of 607 Chinese middle school students (58% female; *M*_age_ = 13.72 years, *SD* = 1.58) participated in the study. A PSIM and PIM were measured by reference to adapted versions of the Growth Mindset Inventory. Two aspects of learning-related outcomes, i.e., learning motivation and academic achievement, were assessed with the Academic Motivation Scale and district-level standardized examinations, respectively. Structural equation modeling (SEM) with bias-corrected bootstrapping was conducted to test the hypothesized mediation model. The results revealed two key findings. First, a PSIM significantly predicted a PIM. Second, a PSIM had different predictive effects on learning motivation and academic achievement through different paths via a PIM. Specifically, a PSIM did not have a significant direct effect on learning motivation, but its effect on learning motivation was significantly and fully mediated by a PIM. Conversely, a PSIM had a significant direct effect on academic achievement, and this effect was not significantly mediated by a PIM. These findings highlight that school-level mindset environments and personal belief systems operate through distinct pathways, suggesting that interventions targeting both institutional climate and individual beliefs may be necessary to effectively enhance students’ motivation and academic performance.

## 1. Introduction

Compared with prior research that has primarily examined the consequences of students’ implicit theories of intelligence at the individual level (He & Zhang, 2024)—referred to here as a personal intelligence mindset (PIM)—little is known about its potential contextual antecedents in a school setting and how this personal mindset impacts students’ learning outcomes under the influence of the school context. Drawing on the perspectives of self-determination theory (SDT; Ryan & Deci, 2000) and mindset theory (Dweck, 1999), we propose that a perceived school intelligence mindset (PSIM), defined as students’ perception of their school’s implicit theory of intelligence and conceptually similar to what prior research has termed an institutional mindset (Murphy & Dweck, 2010), shapes students’ PIM, which in turn influences their learning outcomes. This study focuses on two representative domains: students’ learning motivation and academic achievement. This focus is grounded in both SDT (Ryan & Deci, 2000) and Dweck’s (1999) framework, which together suggest that personal mindsets relate more closely to subjective experiences, whereas contextual signals tend to relate more to objective outcomes. Within this framework, a PIM represents students’ internalized belief about the malleability of intelligence, whereas a PSIM captures the contextual signals in students’ perceptions regarding the broader school climate. We further propose that these two types of intelligence mindset may exert differential predictive effects via different paths. Specifically, a PIM is expected to serve as a stronger predictor of students’ learning motivation than a PSIM, whereas a PSIM is posited as a stronger predictor of academic achievement than a PIM. Moreover, a PSIM is expected to influence learning motivation via the mediating role of a PIM. Through an analysis of these relationships, this study aims to clarify the distinct roles of personal and institutional intelligence mindsets in shaping students’ learning outcomes.

### 1.1. An SDT Perspective on the Roles of a PSIM and PIM in Students’ Learning Outcomes

A personal intelligence mindset (PIM), also known as implicit theories of intelligence at the individual level, refers to individuals’ beliefs about whether intelligence is a malleable trait that can be developed (a growth mindset) or a fixed, innate entity (a fixed mindset; Dweck, 1999). This construct has been extensively studied because of its consequences for students’ academic engagement and resilience. In contrast, a perceived school intelligence mindset (PSIM) refers to an individual student’s perception of the collective beliefs held by the school regarding the malleability and trainability of intelligence. This concept is conceptually aligned with what Murphy and Dweck (2010) termed an institutional mindset, which captures implicit messages about ability and learning embedded in school culture, policies, and common practices. Put differently, a PSIM represents a distinct construct from PIM by tapping into individual students’ perceptions of the institution-level implicit theories of intelligence.

SDT (Ryan & Deci, 2000) provides an insightful theoretical framework for understanding how these two mindsets operate in influencing students’ learning outcomes in school settings. SDT posits that social contexts influence learning motivation, engagement, and performance by either supporting or thwarting individuals’ basic psychological needs for autonomy, competence, and relatedness. A school climate perceived by students as endorsing a growth mindset (i.e., a high PSIM—one that emphasizes effort, learning from mistakes, and the value of improvement—can support the satisfaction of these basic psychological needs. Such a climate supports autonomy by framing challenges as choices for growth, enhances competence through the belief that effort leads to mastery, and fosters relatedness by creating a collaborative rather than a competitively ranked environment. Consequently, such a climate is theorized to facilitate both students’ subjective learning motivation and their objective academic achievement. As Ryan and Deci (2000) stated, “Social contexts that support autonomy, competence, and relatedness facilitate motivation, engagement, and well-being, whereas those that neglect or thwart these needs diminish motivation and undermine performance” (p. 68).

In line with this theoretical claim, consistent findings have shown that perceived contextual mindset is related to students’ learning motivation and academic performance. For example, Canning et al. (2019) found that students who perceived their professors as holding a fixed mindset reported lower levels of learning motivation and achieved lower final grades. Similarly, Muenks et al. (2020) demonstrated that such perceptions were associated with reduced engagement and increased psychological vulnerability, ultimately leading to poorer academic performance. More recently, Kim et al. (2024) also reported that students’ collective perception of instructional mindset played a significant role in influencing their academic outcomes.

Furthermore, SDT (Ryan & Deci, 2000) also provides theoretical insights into the psychological mechanisms that translate a PSIM into students’ learning outcomes. Specifically, the SDT’s principles of internalization and integration offer a theoretical explanation for the translation pathway (Ryan & Deci, 2000). According to this perspective, the relationship between PSIM and PIM can be understood as a process of need-supported internalization. In particular, a school environment perceived as endorsing a growth mindset may support the fulfilment of students’ basic psychological needs for autonomy, competence, and relatedness. When these needs are satisfied, students are more likely to internalize external values and gradually transform perceived contextual beliefs into personally endorsed belief systems. In this sense, a PSIM functions as a contextual precursor that shapes PIM through internalization processes. This suggests that PIM can be understood as the internalized representation of perceived contextual beliefs.

In contrast to the abundant empirical studies on the direct link between a PSIM and students’ learning outcomes, relatively few studies have explored the mediating role of a PIM in this relationship. However, research evidence from organizational psychology may provide empirical support to anticipate this mediating relationship in school settings. For example, Canning et al. (2020) reported that employees who perceived their organization as endorsing a fixed mindset were less likely to internalize the company’s developmental values, and this lack of internalization mediated the negative impact on their job performance in work settings. Similarly, Emerson and Murphy (2015) reported that an organization’s perceived lay theory influenced outcomes such as trust through the mediating role of employees’ stereotype-expectation beliefs. Murphy and Dweck (2010) demonstrated that the effects of an organization’s perceived mindset on participants’ desire to work for the company and their anticipated belongingness were transmitted through the participants’ own internalized self-views. Taken together, these findings suggest that perceived contextual beliefs function through psychological internalization through individual-level belief systems, a process likely to be parallel in educational contexts.

### 1.2. The Different Pathways from a PSIM and a PIM to Learning Motivation and Achievement

Building on the conceptual distinction between contextual and personal belief systems with respect to intelligence, as well as prior evidence of their differential associations with motivational and performance outcomes, it is proposed that PSIM and PIM may not be uniformly related to all aspects of learning outcomes; rather, they may exhibit distinct predictive patterns for subjective psychological aspects (e.g., learning motivation) and objective performance outcomes (e.g., academic achievement). This distinction implies that contextual and personal belief systems operate through different pathways in shaping learning outcomes.

To elaborate, as an internalized and integrated belief system, a PIM operates as a core part of self-determination. It inclines students to focus on the internal and subjective aspects of functioning, such as their personal interpretation of academic challenges, their willingness to exert effort in the face of difficulty, and their intrinsic motivation for mastery learning. In this sense, a PIM is proposed to have a direct link to students’ subjective learning motivation. Conversely, a PSIM, which reflects students’ perceptions of contextual influence, tends to exert its effect by shaping external and objective aspects of functioning, such as the opportunities, incentives, and evaluative standards provided in the school context. In this sense, a PSIM is expected to be more directly related to objective, publicly verifiable outcomes such as grades and standardized test scores (academic achievement), which are closely aligned with institutional structures and evaluative practices. At the same time, although SDT primarily suggests that the influence of contextual beliefs on motivation operates through internalization processes, it remains theoretically plausible that perceived contextual signals may also show a direct association with learning motivation. However, such a pathway is not central to the SDT framework and is therefore treated as exploratory rather than as a primary theoretical prediction in the present study. This reasoning aligns with the ecological view of SDT, highlighting that contextual influence often has a more straightforward impact on objective outcomes that are directly sanctioned by the contextual system (Ryan & Deci, 2000).

This hypothesized differentiation in the pathways through which a PIM and a PSIM affect subjective and objective aspects of learning outcomes is supported by separate strands of empirical research. Studies focusing on the individual level of a PIM consistently show that its stronger correlates are motivational and self-regulatory variables (e.g., Burnette et al., 2013; Liu, 2021). These findings suggest that personal mindset factors are more closely related to subjective motivational experiences than to objective performance outcomes. In contrast, a robust body of research at the contextual level indicates that perceived school or instructor mindsets often show a more direct connection to academic performance. At the contextual level, prior research indicates that perceived school or instructor mindsets are more directly related to academic performance outcomes (e.g., Canning et al., 2019; Park et al., 2018). These findings suggest that contextual cues may exert a more immediate influence on objective performance indicators.

Indeed, prior research has yielded inconsistent findings regarding the relationship between learning motivation and academic achievement. While some studies have documented a significant positive relationship (Legault et al., 2006; Taylor et al., 2014), others suggest that this relationship may be weaker or context-dependent. For example, Trautwein et al. (2006), using reciprocal effects models, found that prior achievement consistently predicted subsequent learning motivation, whereas the reverse path from learning motivation to subsequent achievement was negligible. In a similar vein, Gagné and St. Père (2001) reported that certain self-reported motivational measures did not explain additional variance in academic achievement after controlling for cognitive ability. More recent work has further highlighted the complexity of this relationship (Papageorgiou et al., 2025). These findings suggest that learning motivation and academic achievement represent related but distinct aspects of learning outcomes that may be influenced by different antecedents and mechanisms.

In summary, the theoretical reasoning and empirical findings presented above suggest that a PSIM and a PIM may contribute to student learning outcomes in different roles and through different pathways. Investigating their unique connections to different learning-related outcomes (e.g., learning motivation and academic achievement) separately provides a more precise model of how mindset beliefs operate. This approach implies that fostering comprehensive student growth may require strategies that address both the school climate and students’ personal belief systems.

### 1.3. The Present Study

The preceding sections highlight that PSIM and PIM represent distinct yet interrelated belief systems, which may influence students’ learning outcomes through different mechanisms. While SDT provides a theoretical basis for understanding how contextual beliefs can be internalized into personal belief systems, and prior research suggests differential links between these constructs and subjective versus objective outcomes, several important gaps remain. First, although SDT offers a theoretical rationale for positing a PIM as a mediating mechanism through which a PSIM influences student outcomes, empirical studies explicitly testing this pathway remain scarce. Existing research has predominantly focused on the direct effects of a PIM (Lam & Zhou, 2025) or examined perceived organizational mindset as a contextual predictor (Quaigrain et al., 2026), seldom investigating their dynamic interplay through mediation. Second, limited attention has been paid to the distinct pathways through which these constructs relate to different aspects of learning-related outcomes. Although theoretical reasoning and prior findings suggest that PSIM and PIM may play unique functional roles in subjective versus objective outcomes, this differentiation has rarely been tested within an integrated model.

**Figure 1:**
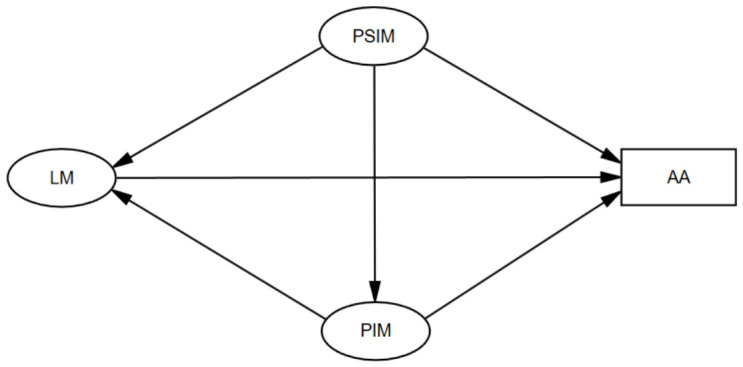
The conceptual model of the present study. Note. PSIM = perceived school intelligence mindset; PIM = personal intelligence mindset; LM = learning motivation; AA = academic achievement. The ovals represent latent variables measured by multi-item scales; the rectangle represents the observed variable of academic achievement.

To address these gaps, the present study proposes and tests a conceptual model (Figure 1) that simultaneously examines the mediating mechanism of a PIM and includes both theoretically grounded indirect pathways and supplementary direct pathways from PSIM to learning motivation and academic achievement. This integrated approach enables us to understand whether the influence of the perceived school context is channelled through personal beliefs and whether each mindset construct uniquely contributes to different facets of students’ learning outcomes. Specifically, we test the following hypotheses:

**Hypothesis 1 (H1).**  *A PSIM positively relates to a PIM*.

**Hypothesis 2 (H2).**  *A PSIM positively relates to learning motivation*.

**Hypothesis 3 (H3).**  *A PSIM positively relates to academic achievement*.

**Hypothesis 4 (H4).**  *A PIM mediates the link between a PSIM and learning motivation*.

**Hypothesis 5 (H5).**  *A PIM mediates the link between a PSIM and academic achievement*.

By testing H1, we assess the role of a PSIM as an antecedent of a PIM. By testing H2 and H3, we assess the differential direct pathways from the contextual predictor (PSIM) to the two outcome variables (learning motivation and academic achievement). By testing H4 and H5, we assess the differential mediating pathways through the personal mediator (PIM) in linking a PSIM and different aspects of learning outcomes.

## 2. Methodology

### 2.1. Participants and Procedures

A target sample of 628 middle school students was recruited from public middle schools in Shanghai, mainland China, to participate in this study. The recruitment process was coordinated with school administrators and class teachers, who distributed study information and online survey links directly to students. Prior to participation, informed consent was obtained from school authorities and parents. Students were informed of the purpose of the study, the voluntary nature of their participation, and the confidentiality of their responses. Participants were given sufficient time to complete the survey during regular class hours under teacher supervision to ensure a standardized testing environment. Following data cleaning, 21 participants were excluded due to incomplete responses (attrition rate = 3.34%), resulting in a final sample of 607 students (58% female). Participants ranged in age from 12 to 15 years (*M*_age_ = 13.72 years, *SD* = 1.58). The sample was drawn from public middle schools located in areas with broadly average socioeconomic backgrounds. These schools follow a standardized national curriculum, providing a consistent academic context across participants. The survey included self-reported measures of a PIM, a PSIM, and learning motivation, and was designed to be concise and age-appropriate. On average, students required approximately five minutes to complete each questionnaire, and the entire data collection process did not exceed 20 min. Academic achievement data were obtained from district-level standardized examinations in core subjects (Chinese, English, mathematics, and science) through official school records to ensure objectivity and accuracy. All procedures adhered to the ethical standards approved by the Human Research Ethics Committee of the authors’ affiliated institution, with strict protocols in place to ensure confidentiality, data security, and voluntary participation.

### 2.2. Instruments

#### 2.2.1. PSIM

A PSIM was assessed on the basis of an adapted version of the 4-item GMI (Dweck, 1999), with item wording systematically modified to reflect students’ perceptions of school-level beliefs about intelligence. Following prior research that contextualized individual mindset items to capture perceived organizational or classroom mindsets (e.g., Murphy & Dweck, 2010; Canning et al., 2019; Muenks et al., 2020), the original items were reworded to reflect students’ perceptions of their school’s collective beliefs about intelligence. For example, the personal item “You have a certain amount of intelligence, and you really can’t do much to change it” was adapted to “At this school, students have a certain amount of intelligence, and they really can’t do much to change it.” Similarly, the item “No matter who you are, you can significantly change your intelligence level” was adapted to “This school is a place where students’ intelligence can be significantly improved.” Responses were given on a 6-point Likert scale ranging from 1 (strongly disagree) to 6 (strongly agree), with higher scores indicating stronger perceptions that the school promotes a growth-oriented climate. Importantly, the adaptation was designed to capture students’ perceptions of school-level beliefs by consistently referring to the school as an institution across all items. This distinction was intended to ensure alignment with the conceptual definition of PSIM as a contextual belief system at the institutional level. Such adaptation has demonstrated good psychometric properties in prior research. Canning et al. (2019) reported high reliability when items were used to measure students’ perceptions of professors’ mindset beliefs (Cronbach’s α = 0.91). Similarly, Muenks et al. (2020) reported consistently strong reliability across four studies (Cronbach’s α = 0.90 in each sample). These findings support the validity of adapting the Implicit Theories of Intelligence Scale to assess perceived contextual beliefs, indicating that such measures reliably capture students’ perceptions of institutional or instructional mindsets. In the present study, the internal consistency of the scale was good, with a Cronbach’s α of 0.80. CFA results indicated an acceptable model fit: *χ*^2^/*df* = 2.47, RMSEA = 0.052, SRMR = 0.041, CFI = 0.94, and TLI = 0.93.

#### 2.2.2. PIM

A PIM was measured on the basis of the Chinese version of the 4-item Growth Mindset Inventory (GMI; Dweck, 1999). The following is an example item: “Even your basic intelligence level can be increased considerably.” Responses were given on a 6-point Likert scale ranging from 1 (strongly disagree) to 6 (strongly agree), with higher scores indicating stronger endorsement of the belief that intelligence is malleable. The GMI also mitigates the positive wording effect noted in recent research (Yu & Kreijkes, 2017), which may otherwise inflate incremental responses due to social desirability, especially among participants familiar with the growth mindset construct (Song, 2018). Prior studies have established the reliability and validity of this scale across diverse contexts, with Cronbach’s α coefficients ranging between 0.76 and 0.88 (C. Hu et al., 2022; Zhao et al., 2023). Its suitability for Chinese educational settings has likewise been confirmed in both teacher (Zhang & He, 2024) and student (Zeng et al., 2016) samples. Moreover, exploratory factor analyses conducted in previous research have demonstrated communalities between 0.59 and 0.72 and factor loadings between 0.77 and 0.85 (Dixson, 2022), providing additional evidence for the structural validity of the instrument. In the present study, the internal consistency of the GMI was satisfactory, with a Cronbach’s *α* coefficient of 0.82. Evidence for structural validity was further provided by a confirmatory factor analysis (CFA), which indicated an adequate model fit: *χ*^2^/*df* = 2.31, RMSEA = 0.048, SRMR = 0.039, CFI = 0.95, and TLI = 0.94.

#### 2.2.3. Learning Motivation

The Academic Motivation Scale (AMS; Vallerand et al., 1992) was used to assess students’ learning motivation. The AMS has been widely adopted across age groups, including in validation studies with middle school populations. It comprises 28 items rated on a 7-point Likert scale ranging from 1 (strongly disagree) to 7 (strongly agree). The instrument captures multiple dimensions of academic motivation: intrinsic motivation to know (e.g., “I go to school because I experience pleasure and satisfaction while learning new things”), intrinsic motivation for accomplishment (e.g., “I study because I enjoy achieving objectives that I set for myself”), intrinsic motivation to experience stimulation (e.g., “I study because I find it thrilling to solve challenging problems”), extrinsic motivation—external regulation (e.g., “I study because I need good grades for my future”), extrinsic motivation—introjected regulation (e.g., “I study because I would feel ashamed if I didn’t do well”), extrinsic motivation—identified regulation (e.g., “I go to school because I think it is important for my future”), and amotivation (e.g., “I don’t know why I go to school, I feel like I am wasting my time”). Items assessing amotivation were reverse-scored, and higher overall scores indicated stronger learning motivation. Previous research has demonstrated good psychometric properties of the AMS, with the Cronbach’s α coefficients generally exceeding 0.80 and confirmatory factor analyses supporting its structural validity (e.g., Guay et al., 2015; RMSEA = 0.068, CFI = 0.95, and TLI = 0.94). In this study, the Academic Motivation Scale exhibited good internal consistency, with a Cronbach’s α coefficient of 0.84. The structural validity of the instrument was further supported by confirmatory factor analysis (CFA), which indicated a satisfactory model fit: *χ*^2^/*df* = 2.48, RMSEA = 0.047, SRMR = 0.038, CFI = 0.95, and TLI = 0.94.

### 2.3. Statistical Analysis

The data were analysed with the assistance of SPSS 30.0 and AMOS 30.0. Descriptive statistics, internal consistency coefficients (Cronbach’s *α*), and Pearson correlations were calculated to examine the preliminary relationships among the study variables. Confirmatory factor analysis (CFA) was conducted to evaluate the measurement model, with model fit assessed by reference to the *χ*^2^/*df* ratio, the RMSEA, the SRMR, the CFI, and the TLI, adopting conventional cut-off criteria (L. T. Hu & Bentler, 1999). To reduce model complexity, the 28 items of the Academic Motivation Scale were aggregated into seven parcels corresponding to its original subdimensions (Little et al., 2002). This parceling approach was adopted to achieve a more parsimonious measurement model and to enhance the stability of parameter estimates. Although the AMS comprises multiple subdimensions, these dimensions can be conceptualized as indicators of a higher-order construct of overall academic motivation, which provides theoretical justification for parceling at the subscale level. In addition, parceling has been shown to improve indicator reliability and reduce sampling error when constructs are well-established. Nevertheless, we acknowledge that parceling may obscure item-level variability and potential multidimensionality, and therefore the results should be interpreted with appropriate caution. Structural equation modelling (SEM) was then employed to test the hypothesized mediating and differential paths. The mediating effect of a PIM was examined on the basis of a bias-corrected bootstrapping approach with 5000 resamples (Preacher & Hayes, 2008). The differences in predictive strength between regression paths were tested by reference to the bootstrap confidence intervals (Cheung, 2009). Missing data (<5%) were addressed through full information maximum likelihood (FIML; Enders & Bandalos, 2001) estimation. Statistical significance was set at *p* < .05.

## 3. Results

### 3.1. Bivariate Analyses

Table 1 displays the bivariate correlations among the study variables. Regarding the study hypothesis, a PSIM was found to be positively and significantly correlated with both a PIM (*r* = 0.42, *p* < .001) and AA (*r* = 0.32, *p* < .001). A PIM was further positively correlated with LM (*r* = 0.36, *p* < .001) and AA (*r* = 0.11, *p* < .05). A small but significant correlation was also observed between a PSIM and LM (*r* = 0.10, *p* < .05). However, LM did not significantly correlate with AA (*r* = −0.02, n.s.). With respect to the demographic variables, age and the number of years of education were strongly and significantly correlated (*r* = 0.81, *p* < .001), whereas neither age nor gender was significantly related to the main study variables.

### 3.2. SEM

#### 3.2.1. Model Fit

**Figure 2:**
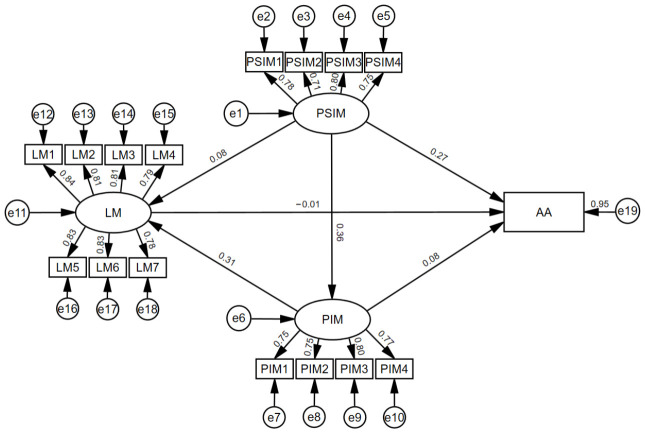
Structural equation model of the mediating role of a personal intelligence mindset in the relationship between a perceived school intelligence mindset and student outcomes. Note. PSIM = perceived school intelligence mindset; PIM = personal intelligence mindset; LM = learning motivation; AA = academic achievement.

The established model (Figure 2) demonstrated a good fit to the data, *χ*^2^(99) = 113.79, *p* = .147, *χ*^2^/*df* = 1.15, RMSEA = 0.016, SRMR = 0.070, CFI = 0.997, and TLI = 0.997. Parsimony-adjusted indices (PNFI = 0.807 and PCFI = 0.823), as well as AIC and ECVI values, further supported the adequacy of the model. Hoelter’s critical *N* exceeded 600 at both the 0.05 and the 0.01 significance levels, thus indicating stability given the current sample size (*n* = 607).

#### 3.2.2. Structural Paths

The results of the structural model are presented in Table 2. The path coefficients (Panel A) revealed that a PSIM significantly predicted a PIM (β = 0.36, *p* < .001), providing support for H1. With respect to the differential direct pathways, the direct path from a PSIM to learning motivation was not significant (β = 0.08, n.s.), indicating that H2 was not supported. Conversely, a PSIM significantly and directly predicted academic achievement (β = 0.27, *p* < .001), supporting H3. In terms of the paths from the mediator, a PIM significantly predicted learning motivation (β = 0.31, *p* < .001) but did not significantly predict academic achievement (β = 0.08, n.s.). The path from LM to AA was also nonsignificant (β = −0.01, n.s.). Bootstrap mediation tests (Panel B) were conducted to test H4 and H5. The results indicated that the indirect effect of a PSIM on LM through a PIM was significant (β = 0.114, 95% CI [0.070, 0.170], *p* < .001), thus supporting H4. In contrast, the indirect effect of a PSIM on AA via a PIM was not significant (β = 0.03, 95% CI [−0.02, 0.08], n.s.), thus rejecting H5. The model explained 32% of the variance in learning motivation and 18% of the variance in academic achievement, indicating a moderate level of explanatory power.

## 4. Discussion

### 4.1. Theoretical Implications

The present study contributes to the literature on growth mindsets by clarifying how a PSIM and PIM are jointly related to students’ learning-related outcomes. The findings hold theoretical significance in the following major respects: they identify a PSIM as a possible antecedent of a PIM, highlight a PIM as a mediating mechanism, and disentangle the differential roles of a PSIM and PIM. Crucially, the specific pattern of supported and unsupported hypotheses provides nuanced insights into the distinct roles and pathways through which these personal and contextual mindsets operate.

First, this study advances the theorization of the antecedents of a PIM. While prior research has primarily emphasized a PIM as a personal belief system predicting educational outcomes (e.g., He & Zhang, 2024), more recent work has begun to highlight the importance of contextual and institutional influences in shaping students’ belief systems (e.g., Bardach et al., 2024; Kim et al., 2024). The present findings extend this emerging line of research by suggesting that students’ personal beliefs may be partly shaped by their perceived contextual mindset at school with respect to intelligence. In line with SDT (Ryan & Deci, 2000), the results are consistent with the idea that students internalize and integrate the values that they perceive in their school climate. When students perceive their school as promoting effort and improvement, they may be more likely to endorse a personal growth mindset. This interpretation aligns with organizational studies showing that a perceived contextual mindset in the workplace can be associated with employees’ self-views and downstream outcomes (Canning et al., 2020; Emerson & Murphy, 2015; Murphy & Dweck, 2010). Extending such insights to the educational context, the present study suggests that a PSIM could be viewed as a plausible antecedent of a PIM, offering a way to improve the understanding of how personal intelligence beliefs may develop through school-level perceptions. This perspective also resonates with the broader question of how socialization processes may shape individual-level beliefs: the findings imply that a PIM should not be treated as entirely dispositional and, instead, should be treated as situated and potentially malleable, reflecting interactions between individual agency and contextual cues.

Second, the non-significant direct path from a PSIM to learning motivation (H2 not supported), coupled with the significant mediation through a PIM (H4 supported), provides a refined understanding of how the school mindset influences motivation. This pattern is consistent with the theoretically indirect nature of the relationship between contextual beliefs and motivation. From an SDT perspective, contextual influences are typically internalized through psychological processes before shaping motivational outcomes. Accordingly, the effect of PSIM on learning motivation appears to operate primarily through PIM rather than through a direct pathway. Notably, the significant bivariate correlation between a PSIM and learning motivation (*r* = 0.10, *p* < .05) became a nonsignificant direct path in the SEM analysis when a PIM was included, indicating that this relationship is largely accounted for by PIM. This finding is consistent with SDT’s emphasis on internalization as a key process through which external values become personally endorsed and motivationally potent (Ryan & Deci, 2000). It also suggests that PSIM and PIM represent only part of a broader set of factors influencing students’ learning motivation and academic achievement.

Third, the pattern of mediation findings offers empirical support for the theorized distinct pathways. The significant direct path to academic achievement (H3 supported) versus the nonsignificant mediation for achievement (H5 not supported) lends support to the theoretical claims distinguishing between personal and contextual predictors. It suggests that a PIM serves as a crucial psychological conduit for motivational outcomes but is not a necessary conduit for performance outcomes. This finding indicates that the impact of a PSIM on academic achievement may operate through contextual mechanisms that directly facilitate or constrain performance outcomes. This dissociation emphasizes the claim that a PSIM and PIM are not interchangeable and, instead, have unique functional roles in learning-related outcomes, with PIM contributing more to the internal world of learning motivation and a PSIM contributing more to the external metric of academic performance and achievement.

The results further underscore the potential importance of a PIM as a mediating mechanism through which a perceived contextual mindset relates to students’ motivational states. The significant indirect effect of a PSIM on learning motivation via a PIM (H4 supported) is consistent with SDT’s argument that values from the environment may become influential when internalized into the self (Ryan & Deci, 2000). Previous research has documented direct associations between a PSIM and motivation and performance (Canning et al., 2019; Kim et al., 2024; Muenks et al., 2020), but few studies have explicitly tested a PIM as a mediator. By showing that students’ PIM may serve to translate perceived school-level beliefs into motivational outcomes, this study adds nuance to prior debates on whether growth mindset interventions should target individuals or institutions. The findings suggest that contextual signals may exert their influence on motivation primarily when they are internalized into personal belief systems rather than remaining at the perceptual level. Theoretically, this implies that contextual beliefs may not operate in isolation but could be psychologically filtered through individual belief systems.

This study refines theoretical accounts by clarifying the differential predictive paths of a PSIM and a PIM. Consistent with SDT, the results suggest that as an internalized belief system, a PIM was a significant predictor of students’ subjective motivational experiences, whereas as a perceived contextual mindset, a PSIM was a significant predictor of objective achievement outcomes. This distinction complements findings that personal implicit theories tend to show stronger links to motivational processes than to grades (Burnette et al., 2013; Liu, 2021), while perceived classroom or school mindsets are often linked to performance indicators (Canning et al., 2019; Park et al., 2018). By modelling both together, this study provides evidence for what might be considered a dual-pathway perspective: a PIM is more strongly related to learning motivation, whereas a PSIM is more strongly associated with academic achievement. This framework moves the field beyond single-path models and emphasizes the differentiated contributions of personal and perceived contextual mindsets. Importantly, this perspective also allows for further theorization of how subjective and objective outcomes may be jointly produced. For instance, motivation may function as a proximal outcome of a PIM, whereas achievement may reflect the cumulative influence of multiple contextual factors beyond individual belief systems.

Finally, the study contributes to the broader theoretical integration of individual and perceived contextual perspectives in mindset research. Previous studies have often focused exclusively on either a PIM (Lam & Zhou, 2025) or a perceived contextual mindset (Quaigrain et al., 2026). By examining their interplay, this study reveals that a PSIM is related to a PIM (H1 supported), that a PIM mediates the relationship between a PSIM and learning motivation (H4 supported), and that both exhibit distinct yet complementary predictive roles. Furthermore, the findings suggest that alignment between personal and perceived contextual mindsets is crucial, as incongruence may weaken motivational outcomes, whereas alignment strengthens both internalization and performance (Giel et al., 2020; Park et al., 2018). This integration suggests that students’ learning is shaped not only by what they personally believe but also by how they interpret the mindset climate of their school. Therefore, the findings point towards a more ecological theorization of mindset, in which personal and perceived contextual mindsets are viewed as mutually constitutive elements of the learning process. Such theorization underscores that individual-level and context-level processes should not be conceptualized as independent, but rather as dynamically co-constructed within the learning process. At the same time, the relatively modest effect sizes observed in this study suggest that these processes operate alongside a broader range of factors influencing students’ learning outcomes.

### 4.2. Practical Implications

The findings of this study provide several practical implications for schools, teachers, and policymakers by indicating how different aspects of the school environment and students’ personal beliefs can be leveraged to support learning motivation and academic achievement.

First, the finding that a PSIM significantly predicts a PIM (H1 supported) highlights the importance of the institutional environment as an antecedent for personal belief development. For schools aiming to cultivate a growth mindset among students, the initial focus should not be solely on individual interventions; rather, schools should actively align their culture, administrative policies, and instructional practices to consistently communicate a growth-oriented message. This system-level alignment, which is consistent with the concept of establishing an institutional mindset, tends to be the foundational step for shaping students’ personal beliefs (Murphy & Dweck, 2010). This emphasis on the institutional context is essential because social environments are fundamental to shaping individual belief systems (Ryan & Deci, 2000).

Second, the direct and unmediated effect of a PSIM on academic achievement (H3 supported, H5 not supported) points to a potentially more straightforward path for improving performance outcomes. This pattern suggests that schools can directly influence academic performance by adjusting institutional policies and structural factors that shape the perceived school climate, such as assessment methods that reward improvement, recognition systems that highlight effort, and a school-wide discourse that avoids labelling students based on innate talent, which may have a direct effect on student grades and test scores. Therefore, school leaders can invest in shaping these broader systemic and cultural elements, akin to the institutional mindset described by Murphy and Dweck (2010), with the expectation of a potential direct return on academic performance. This creates a dual-pronged approach: one set of strategies (focusing on internalization) targets the development of sustained motivation, while another set (focusing on the environment) targets the facilitation of immediate academic performance.

Third, the finding that a PSIM influenced learning motivation only through the mediation of a PIM (H4 supported) and not via a direct path (H2 not supported) underscores a critical implication: simply creating a growth-minded school climate is not enough to directly enhance students’ motivation; the climate is best utilized when it is actively internalized by students. This finding aligns with SDT (Ryan & Deci, 2000), which posits that external values tend to become influential when internalized into the self. This suggests that schools should move beyond simply promoting growth-minded messages and instead create concrete classroom conditions that actively support students in internalizing these messages. Teachers play a pivotal role in this process. Instead of just telling students that they can grow, teachers should employ strategies that may facilitate internalization, such as providing effort-based feedback, modelling adaptive responses to setbacks, and encouraging mastery-oriented goals, all of which are concrete practices suggested by mindset theorists (Dweck, 1999, 2017). Prior evidence supports the importance of such internalization. For instance, Zeng et al. (2016) reported that a growth mindset was linked to Chinese students’ engagement and well-being via resilience, highlighting that personal belief may be the active ingredient for positive psychological outcomes. This finding suggests that schools’ growth-oriented climates are not just perceived; rather, they are transformed into students’ personal beliefs, which in turn may drive their motivation to learn.

Finally, the clear dissociation between the pathways to motivation and achievement implies that interventions might need to be multi-faceted to comprehensively support students. Relying solely on student-level mindset workshops may improve motivation—as personal mindset factors are more tightly linked to motivational experiences (Burnette et al., 2013; Liu, 2021)—but might not sufficiently impact achievement if the broader perceived school climate remains fixed oriented (Canning et al., 2019). Conversely, only reforming school-level policies might increase grades without fostering the intrinsic motivation that supports long-term engagement. Therefore, the most effective approach would tend to integrate both levels: teacher professional development should focus on pedagogical practices that support the internalization of a growth mindset, while school leadership simultaneously works to align institutional policies and the broader school culture with growth-oriented principles (Bardach et al., 2024; Quaigrain et al., 2026). This approach can help ensure that students are not receiving conflicting signals and that supportive environments and empowered individuals may mutually reinforce each other for optimal development. However, these recommendations should be interpreted with caution given the modest magnitude of some observed effects.

## 5. Limitations and Directions for Future Research

The first limitation lies in the scope of the mediation model. In this study, a PIM was modelled as the only mediator between a PSIM and student outcomes. While this design was theoretically informed and successfully established a PIM’s specific mediating role in learning motivation, it may underestimate the complexity of the psychological processes that link perceived school-level beliefs to academic achievement. Given that a PSIM predicted AA directly rather than through a PIM, other factors, such as self-efficacy (Burke et al., 2009), resilience (Satterwhite & Luchner, 2016), or psychological needs satisfaction (Bozhani et al., 2025), could act as additional mediators or moderators of this direct pathway. Therefore, future studies should broaden the model by incorporating these constructs, which would allow for a more complete understanding of how perceived contextual beliefs are internalized into motivation versus how they directly facilitate performance. In addition, although the sample was adequate for the analyses conducted, future research could benefit from more diverse samples to enhance the generalizability of the findings.

The second limitation relates to the measurement of learning motivation. The Academic Motivation Scale used here is a well-validated tool, but as a self-report measure, it may not directly capture behavioural persistence or effort in real academic settings. This limitation may partly explain why learning motivation, despite being a robust outcome of a PIM, did not significantly predict academic achievement in the model. Future research could combine self-reports with behavioural indicators such as persistence with challenging tasks, the time invested in study, or longitudinal tracking of course engagement. Recent systematic reviews in educational psychology have emphasized the value of such multi-method approaches, showing that integrating self-reported measures with behavioural data (e.g., log files and course participation records) provides a more comprehensive account of students’ learning processes (Papageorgiou et al., 2025). Therefore, integrating subjective and objective measures would yield a more precise understanding of how mindsets influence both motivational experiences and performance outcomes.

The third limitation concerns the cross-sectional design, which offers only a static snapshot of the relationships among a PSIM, a PIM, and learning outcomes. Although the hypothesized model was supported in part, it is not possible to establish temporal precedence or reciprocal influences. For instance, the process of internalization—whereby a PSIM shapes a PIM—likely unfolds over time. Similarly, students’ personal mindsets may evolve in response to prior achievement, or perceptions of school ethos may shift after repeated experiences of success or failure. To overcome this challenge, future studies should adopt longitudinal designs that follow students across academic terms or critical educational transitions. Such approaches would clarify whether perceived contextual beliefs drive changes in personal beliefs and outcomes over time or whether there exist feedback loops between performance and perceptions of the school climate. Evidence from recent longitudinal studies in educational psychology has demonstrated the advantages of this approach, showing how baseline educational factors can predict subsequent psychological outcomes over time (Tian & Tang, 2025).

The fourth limitation relates to the operationalization of a PSIM. In this study, a PSIM was assessed as a generalized perception of the overall school climate. However, students’ experiences are often situated in specific classrooms or subject areas, and perceived classroom culture may vary considerably across teachers and disciplines (Eccles & Roeser, 2011). Such variation implies that a PSIM is not necessarily monolithic; rather, it is experienced through multiple micro-contexts that could differentially contribute to personal beliefs. Therefore, future research could examine subject-specific or teacher-specific perceptions to clarify whether the observed pathways—particularly the direct effect on achievement and the fully mediated effect on motivation—are consistent across different domains of learning.

The fifth limitation pertains to the absolute magnitude of the total effect in the PSIM → LM pathway (total effect = 0.19), despite the path being successfully and fully mediated by a PIM. While this pattern supports the theoretical necessity of internalization, the literature suggests that this finding may be a consequence of the weak overall association. Specifically, as Ledermann et al. (2025) noted, “full statistical mediation is more likely to occur when the total effect is small” (p. 3). This phenomenon suggests that the absolute influence of the established mechanism is modest. Although the robustness of the indirect effect (indirect effect = 0.11) was confirmed, future studies should aim to increase the explanatory power of this link. This task could involve utilizing domain-specific intrinsic motivation as a more precise outcome measure or employing models that meticulously control for crucial cognitive covariates, thereby potentially amplifying the observed effect size.

## 6. Conclusions

This study examined how a PSIM relates to students’ PIM and their learning outcomes. The findings demonstrate that a PSIM is positively associated with a PIM, establishing the perceived school context as a significant antecedent of personal beliefs. Furthermore, the influence of a PSIM on learning motivation was fully mediated by a PIM, underscoring the critical role of internalization in shaping students’ motivational states. In contrast, a PSIM exerted a direct effect on academic achievement, indicating that its impact on objective performance operates through pathways that are distinct from personal belief internalization. These results contribute to clarifying the distinct pathways through which perceived contextual and personal mindsets are related to student outcomes and suggest that both levels deserve integrated attention in theory and practice. While the evidence points to plausible processes through which perceived school intelligence mindsets shape individual beliefs and outcomes, further longitudinal and multi-method research is needed to firmly establish the underlying temporal mechanisms and to examine additional mediators or moderators. More importantly, this study contributes to the literature by demonstrating that contextual and personal mindsets operate through distinct pathways to different learning outcomes. While personal mindset primarily relates to students’ internal motivational processes, perceived contextual mindset shows a more direct association with externally evaluated performance outcomes. This dual-pathway perspective advances existing theories by moving beyond single-path explanations and offering a more differentiated account of how belief systems function within educational contexts.

## Figures and Tables

**Table 1:** Descriptive statistics, reliability estimates and intercorrelations of the study variables.

Variables	Mean (*SD*)	1	2	3	4	5	6	7
1. Age	13.72 (1.58)	-						
2. Gender	-	0.01	-					
3. Edu	7.84 (1.36)	0.81 ***	0.03	-				
4. PSIM	2.58 (0.43)	0.05	0.02	0.04	(0.80)			
5. PIM	4.02 (0.62)	0.05	0.02	0.02	0.42 ***	(0.82)		
6. LM	4.53 (0.81)	0.04	0.03	0.02	0.10 *	0.36 ***	(0.84)	
7. AA	78.15 (13.57)	0.06	0.02	0.03	0.32 ***	0.11 *	−0.02	-

Notes. *n* = 607. Edu = number of years of education; PSIM = perceived school intelligence mindset; PIM = personal intelligence mindset; LM = learning motivation; AA = academic achievement. The diagonal values in parentheses represent the alpha reliability coefficients. * *p* < 0.05, *** *p* < 0.01.

**Table 2:** Direct, indirect, and total effects in the structural equation model. (Panel A) Standardized and unstandardized path coefficients. (Panel B) Bootstrap mediation.

(Panel A)				
**Pathway**	**β**	**B**	**S.E.**	***p***
PSIM → PIM	0.36	0.51	0.08	<.001
PIM → LM	0.31	0.22	0.04	<.001
PSIM → LM	0.08	0.08	0.05	n.s.
PSIM → AA	0.27	3.60	0.62	<.001
PIM → AA	0.08	0.74	0.41	n.s.
LM → AA	−0.01	−0.11	0.58	n.s.
(Panel B)				
**Indirect Pathway**	**Total Effect (β)**	**Direct Effect (β)**	**Indirect Effect (β)**	**95% CI**
PSIM → PIM → LM	0.19	0.08	0.11	[0.07, 0.17]
PSIM → PIM → AA	0.30	0.27	0.03	[−0.02, 0.08]

Notes. PSIM = perceived school intelligence mindset; PIM = personal intelligence mindset; LM = learning motivation; AA = academic achievement.

## Data Availability

The data presented in this study are available on request from the authors due to privacy or ethical restrictions.
